# Response to the Letter to the Editor regarding “Comparison of temporalis fascia muscle and full-thickness cartilage grafts in type 1 pediatric tympanoplasties” by Yegin et al. (Braz J Otorhinolaryngol. 2016;82:695–701)^☆^

**DOI:** 10.1016/j.bjorl.2017.02.007

**Published:** 2017-03-08

**Authors:** Yakup Yegin, Mustafa Çelik

**Affiliations:** Bakırköy Dr. Sadi Konuk Training and Research Hospital, Department of Otorhinolaryngology – Head and Neck Surgery, Istanbul, Turkey

Dear Editor,

We would like to thank Dr. Zheng-cai Lou for valuable and precise comments on our article.^1^ Firstly, the main outcomes of type 1 pediatric tympanoplasty are the graft success rates and postoperative hearing outcomes. Our results indicate that the graft success rate was 92.1% of the cartilage group compared with 65.0% of the temporal fascia group, respectively. In the fascia group, preoperative ABG was 33.68 ± 11.44 dB and postoperative ABG was 24.25 ± 12.68 dB. In cartilage group, preoperative ABG was 35.68 ± 12.94 dB and postoperative ABG was 26.113 ± 12.87 dB. The anatomical success rate of cartilage group was significantly better than fascia group (*p* < 0.01). There was no significant difference among functional outcomes between fascia and cartilage groups (*p* > 0.05). The thickness of tragal cartilage was accurately measured by a micrometer and recorded intraoperatively. Regular whole-length bar of tragal cartilage was excised and the thickness of tragal cartilage was measured. Measurement of thickness was performed by the same surgeon (YY). All measurements were repeated by the second surgeon (MÇ) to avoid inter-observer variations. Three measurements were performed to avoid discrepancy and incorrect results. Measurements were consisting of superior, middle and inferior part of tragal cartilage. The average thickness of tragal cartilage was accepted as average three measurements. The total average thickness of tragal cartilage was 0.693 ± 0.094 mm in males and 0.687 ± 0.058 mm in females. To our knowledge, the present study is the first study of measuring thickness of tragal cartilage in pediatric tympanoplasty.^2^

Dr. Zheng-cai Lou said that “We believed that a “retrospective review” and “randomly allocated” are contradictory.” for comments on our study design. Of course, you are right. But, in discussion, explanation of this condition was putted in an appearance. Honestly, there is no consensus on the selection of graft materials for tympanoplasties; it depends entirely on surgeon experience and preferences. In our clinic, the selection of graft materials for pediatric tympanoplasties depends on entirely on surgeon experience and preferences. It means randomly allocated to surgery using temporalis fascia muscle or tragal cartilage grafts by the surgeons. It is not for our study design, in terms of selection of the graft materials in general. Therefore, for this condition, there was no contradictory. We think you could understand us better if carefully. We are agree with you about the further prospective studies, with random control, a larger sample size and longer follow-up are needed to compare the anatomical and functional outcomes of various cartilage types.

For your other comments on exclusion criterias, you are right and we would like to thank you for bringing this to our attention. Granulation tissue may affect the success of pediatric tympanoplasties, but no studies have reported about this condition. Honestly, in our patient's chart, we have not recorded about granulation tissue for pediatric tympanoplasties.

Although data on the selection of graft materials for pediatric tympanoplasties continue to rise, there is no consensus on the selection of graft materials for tympanoplasties for now.^3^ However, we have also planned to compare the anatomical and functional outcomes of various graft types (pericondrium grafts, fascia grafts, various cartilage graft [conchal and tragal cartilages]) and differing thicknesses of cartilage grafts in pediatric tympanoplasties in future.

Best regards.

## Conflicts of interest

The authors declare no conflicts of interest.

## Financial disclosure

The authors declare that this case has received no financial support.
